# Toward an Event-Based and Quality-Assured Air Sampling: A Portable System for Sensing and Sampling Volatile Organic Compounds

**DOI:** 10.1021/acs.analchem.5c03799

**Published:** 2025-10-22

**Authors:** Thomas Mayer, Tobias Goblirsch, Ralf Petrich, Helko Borsdorf

**Affiliations:** † Department Monitoring and Exploration Technologies, Field Analytical Chemistry Group, UFZ-Helmholtz Centre for Environmental Research, Permoserstraße 15, 04318 Leipzig, Germany; ‡ IFU GmbH Private Institute for Analytics, An der Autobahn 7, 09669 Frankenberg/Sa, Germany

## Abstract

Sensors for monitoring volatile organic compounds are commonly employed for air quality control in indoor and outdoor environment. They provide data with high temporal and spatial resolution. Although these sensors can detect possible concentration changes very quickly, they do not provide any information about the composition of air pollutants. Furthermore, the measurements are strongly influenced by gaseous interfering compounds and meteorological conditions. It is necessary to carry out measurements in the laboratory for reliable and credible data. The adsorptive enrichment of air samples on solid adsorbents is the most commonly used method for taking air samples. We wanted to combine the advantages of both approaches. Therefore, a portable modular sensor and multitube sequential sampling system has been developed. It combines real-time online monitoring of ambient air using a gas sensor array and quality-assured active sampling. The sensor module consists of an optimized 3D-printed sensor chamber where up to 4 gas sensors can be integrated. The sampler module was equipped with up to 32 commercially available sorbent tubes. The inlet pressure of the mass flow controller and temperature are permanently recorded, allowing the detection of a leakage or tube clogging during the sampling procedure. The remote access to the data from both the sensors and the sampler parameters allows the identification of possible malfunctions, which limits the necessary presence of an operator during long-term measurements. The power supply is realized with a lightweight battery, which can be charged by solar panels.

## Graphical abstract

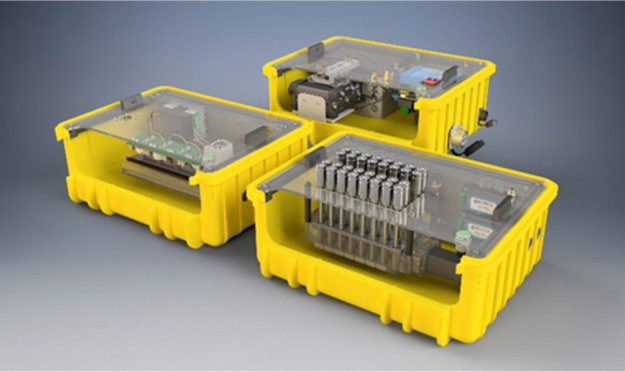

## Introduction

Volatile and semivolatile organic compounds (VOCs and SVOCs) are released into the atmosphere from biogenic and anthropogenic sources. Anthropogenic sources result from human activities including fossil fuel combustion and industrial processes, industrial emissions, or emissions from motor vehicles.^1^ Biogenic volatile organic compounds (BVOCs) are biosynthetically formed within plants via two distinct metabolic pathways, termed the mevalonic acid pathway, which takes place in the microsomes and cytoplasm, and the methylerythritol phosphate pathway, which takes place in chloroplasts.^2^ Therefore, vegetation emits significant amounts of VOCs (isoprene, terpenes, terpenoids, and sesquiterpenes) into the atmosphere, influencing ecological interactions and atmospheric chemistry.^3^ Independent of their source, all VOCs are of concern due to their environmental and human health impacts. A necessity for the analysis of airborne pollutants is also the detection of chemical hazardous gases including chemical warfare agents (CWAs) for the protection of public places and critical infrastructures as well as toxic industrial chemicals (TICs) by first responders.^4^

There are various established offline analytical methods for determining the VOCs. Gas chromatography (GC) is mainly used in combination with mass spectrometry (MS) for the identification of VOCs. These analytical techniques are comprehensively characterized. Methods for calibration and quality assurance are available and easy to handle. Consequently, these analytical methods provide very reliable and reproducible results, and it has been demonstrated that concentrations at levels as low as pg L^–1^ can be detected. However, it requires sampling and the transportation of samples to a lab. In contrast to analysis, much less attention is focused on the accuracy of air sampling, although the sampling procedure is a crucial and possibly the most significant event during the analysis of VOCs from ambient or indoor air.

The adsorptive enrichment on solid adsorbents is the most widely used sampling approach. The preconcentration of samples is necessary for determining the low levels of VOCs. The air samples are sucked via a suitable pump through a sorbent tube containing porous polymers (e.g., TENAX) or graphitized carbon black (e.g., Carbograph) or a combination of different adsorbents (multibed sorbent tubes).^5,6^ This active sampling is often carried out using simple, adjustable diaphragm pumps without controlling the actual volume flow, and only one sorbent tube can be loaded. Although progress regarding automation, miniaturization, and quality assurance in sampling has been reported in the literature in recent years, we have not found a multitube sequential sampler with remote monitoring of the operating parameters, which permits an unsupervised long-term operation on-site. Portable, low-cost samplers for distributed sampling of VOCs equipped with one sorbent tube and the possibility to store all important parameters (sample volume, start time, temperatures) on an onboard SD card were recently described.^7^ Furthermore, desorption units for micro-thermal sorbent tubes were developed for coupling to hyper-fast gas chromatography.^8^ The miniaturization of samplers (with just a few sorbent tubes) is also the focus of current developments in order to mount them on drones.^9,10^

In addition to these conventional active air sampling techniques, miniaturized enrichment techniques like needle trap microextraction (NTME), in-tube extraction (ITEX), solid-phase microextraction (SPME), or stir bar sorptive extraction (SBSE) were used for special applications. However, mainly qualitative results are described. It is still difficult and time-consuming to calibrate these techniques.^11^

Passive air samplers do not require any equipment such as pumps or a power supply. The analytes migrate from ambient air to the sampler through molecular diffusion. These samplers consist of an adsorbing cartridge (mainly activated charcoal), which is placed inside of a diffusive body (e.g., microporous polyethylene or silicone membranes). Their quantitative interpretation is often only known with high uncertainty due to different uptake rates for different substances and their dependence on meteorological parameters.^12^

If the concentrations of compounds of interest are sufficiently high, online methods such as online GC with a photoionization detector or flame ionization detector can be used or the samples can be transferred into the lab using canisters or sampling bags (whole-air sampling).^13^ However, these sampling techniques can be performed only manually and cannot be automated. The online analytical techniques require a power and gas supply onsite.

Field deployable analytical methods for VOCs can monitor and track air quality, identify sources of pollution, and support efforts to reduce environmental exposure. Low-cost VOC sensors permit a near-real-time observation. The most commonly used sensor types for VOC monitoring are photoionization detectors (PID), electrochemical sensors (ECS), metal oxide sensors (MOS), pellistors, surface acoustic wave sensors (SAW), and quartz crystal microbalances (QCM).^14^ Portable gas sensor systems are typically configured as sensor array with multiple gas sensors.^15^ Such low-cost air quality sensors are often integrated in sensor networks and provide data in high temporal and spatial resolution.^16^ They are smaller in size, significantly cheaper, and more portable in comparison to standard instruments used for VOC monitoring. Nevertheless, low-cost sensors are limited in terms of sensitivity, selectivity, and reliability. It is nearly impossible to assign the sensor signals to the concentrations of substances, especially when unpredictable combinations of VOCs occur or sites with a rapidly changing composition are investigated, as is the case in real-world applications.^17^

In the presented monitoring platform, we combine real-time instrumentation and well-established offline analytical techniques after the adsorptive enrichment of air samples for providing credible analytical data. This unit was developed for monitoring, sensing, and assessing environmental exposures and hazards. Low-cost VOC sensors permanently monitor the environment. If there is an increase in the signal at the sensors, the active air sampling is started automatically. It is therefore possible to identify which substances caused the change in the sensor signal. However, the complex and expensive laboratory analysis is used only if the sensor signals differ from those of ambient conditions. In our present configuration, we developed a sensor module that can be equipped with four commercially available sensors. Therefore, the sensors can be configured by the user according to a special application. Based on our own experience with commercial samplers, a key focus in the development of the air sampling unit was to ensure a constant sample volume flow during the entire sampling period. Therefore, all relevant parameters (temperature, humidity, sample volume, inlet pressure of the mass flow controller) are monitored in real time, saved, and transferred to the user via remote access. Technical problems can thus be detected immediately. The configuration as an IoT device permits the networking of different monitoring platforms. The complete system was developed as a battery- and solar-powered unit for long-term onsite application.

## Materials and Methods

### Functionality of the System

The system consists of three modules: sensor module, sampler module, and power module. Each module is housed in a separate outdoor case (B&W Outdoor case type 3000, Ibbenbüren, Germany, 365 mm length, 295 mm width, 170 mm depth, 11.7 L volume). The weatherproof cases and the plugs and connections between the modules are all IP66-rated. The modified cases for the sensor and sampler module have been classified as IP42 due to the integration of air inlets. The air inlet and outlet ducts are covered with woven stainless-steel wire meshes of an aperture size of 0.42 mm. Furthermore, the samples are protected against dust by the use of the DiffLok caps and the ozone filters.

The purpose of coupling the sensor and sampling module is to realize event-based sampling. The ambient air is permanently monitored by the sensors. The sampling procedure is then automatically started when the adjustable threshold values of the sensor signals are exceeded. This function can be particularly useful for monitoring industrial sites, industrial accidents, or the detection of airborne chemical hazards including chemical warfare agents. When multiple systems are employed, sampling can also be started simultaneously at different sampling sites if measurements with spatial resolution are required. This function is realized by the integration of a gateway and the associated Internet capability of the system in conjunction with our control software and permits the operation of several samplers as an observation network. Furthermore, the sampling can be started at a designated date and time or manually. The combination of sensor technology and sampling also provides additional information for long-term environmental monitoring. The configuration as multitube sequential sampler with the possibility of loading the sorbent tubes one after the other permits measurements with high temporal resolution. It is known from literature that active sampling of VOCs is sometimes influenced by on-site conditions, such as humidity or interfering compounds.^18^ For example, the occurrence of BVOCs and their sampling can be affected by the concentrations of ozone and nitrogen oxides.^19^ Using the sensor module, these gases can be monitored during the sampling period and provide complementary data for the interpretation of BVOC concentrations.

A key benefit of the system is the quality assurance of the sampling procedure by determining and recording the exact sample volumes during the sampling procedure. Leakages in the sample path or tube clogging can be detected by recording the inlet pressure of the mass flow controller. Humidity and temperature are also permanently logged.

The power module is equipped with a rechargeable battery (Yuasa Cyclic VRLA Battery REC14–12 (12 V 14Ah), Kyo̅to, Japan) and a solar charge controller (MPPT 75/10, Victron Energy, Almere, Netherlands), enabling solar-powered charging via solar panels (WS80SF foldable solar panel 80 W, 12 V, Wattstunde, Lüneburg Germany). This facilitates autonomous operation at sites without an external power connection. The module also features a single-board computer (Raspberry Pi 3 Model B, Raspberry Pi Foundation, Cambridge, UK) and an LTE router (RUT 955, Teltonika IOT Group, Vilnius, Lithuania). The control software, programmed in Python, is located on the integrated computer and can be accessed as a web interface via the Internet or the local WLAN. This interface enables system configuration for specific applications, including time- or event-based sampling, and displays and logs all sensor data recorded during the sampling procedure in a log file.

For specialized applications where simultaneous sensor and sampling module operation are not required, both modules can be applied separately in conjunction with the power module.

### Hardware Description of the Sensor Module

In its current configuration, the sensor module is designed for the detection of BVOC emissions from plants and is therefore equipped with an electrochemical VOC sensor (VOC–B4), a PID sensor (PIDX-A-004) for VOCs, a nitrogen dioxide sensor (NO2–B43F), and an ozone + nitrogen dioxide sensor (OX-B431). All sensors were obtained from Alphasense (Great Notley, UK). The sensors are used with the corresponding evaluation boards (4-electrode individual sensor board ISB, Alphasense), which provide a standardized output voltage of 0–5 V. The signal processing board for each sensor was developed on the basis of the ISBs. This is necessary for providing the appropriate voltage and power as well as to convert the analogue signals into digital signals. The signal conversion process is executed independently on each sensor using an analog-to-digital converter (ADS1110, Texas Instruments Inc., Dallas, USA). This configuration is designed to minimize interference and to enable the easy replacement of the sensors. The communication process is facilitated by an I2C interface. The sensors are placed in a self-developed sensor chamber with a length of 180 mm. It was manufactured from COC (cyclic olefin copolymer) using a 3D printer (S3, Ultimaker, Geldermalsen, Netherlands). COC was used due to its thermal stability, low water absorption, and low off-gassing. Ambient air is directed into the case of the sensor module via a radial fan (Sunon EF50151B3, Sunonwealth Electric Machine Industry Co., Ltd., Kaohsiung, Taiwan) mounted on the inside of the case. To ensure a constant air flow of 700 mL min^–1^ through the sensor chamber, a MF15B axial fan (SEPA Europe GmbH, Eschbach, Germany) was positioned at its gas outlet. The sensor chamber is enclosed in an aluminum body with a 5 mm thickness with a self-regulating PTC (Positive Temperature Coefficient) heater on its surface (HP06-2/04-24, Farnell, Leeds, UK).

The optimization of gas flows within the sensor chamber was carried out using the Computational Fluid Dynamics (CFD) software Autodesk CFD 2024 (Autodesk Inc., San Francisco, USA). For this purpose, two designs of the sensor chamber were created by using Autodesk Inventor Professional 2024. The first design was analogous to a commercially available gas hood (Alphasense GSHD-00), with the difference that 4 sensors can be installed in series. The inner diameter of the flow path is 7.5 mm and increases to 19 mm in the regions of the sensors.

The second design was equipped with swirl plates to generate turbulences in order to reduce the boundary layer on the sensor surface, ensuring the most efficient substance transfer. Table 1 shows the averaged values of various flow parameters at a distance of 0.5 mm from the sensor surface.

**Table 1:** Flow Parameters of Different Sensor Chambers

	average velocity [m s^–1^]	turbulent kinetic energy [m^2^ s^–2^]
design 1	0.12	<0.006
design 2	0.46	31.26

The integration of the swirl plates leads to a higher mass transfer rate at the sensor surface, as evidenced by the flow velocity, as a measure of convective transport, and the turbulent kinetic energy, as an indicator of the intensity of turbulence.

The flow situation shown in Figure 1 illustrates the facts described above. The analysis of the flow lines suggests that in Design 1 of the sensor chamber (Figure 1A), only a small number of turbulences occur at the sensor surfaces. In contrast, Design 2 (Figure 1B) significantly increases the flow and turbulence in the direction of the sensor surfaces.

**Figure 1:**
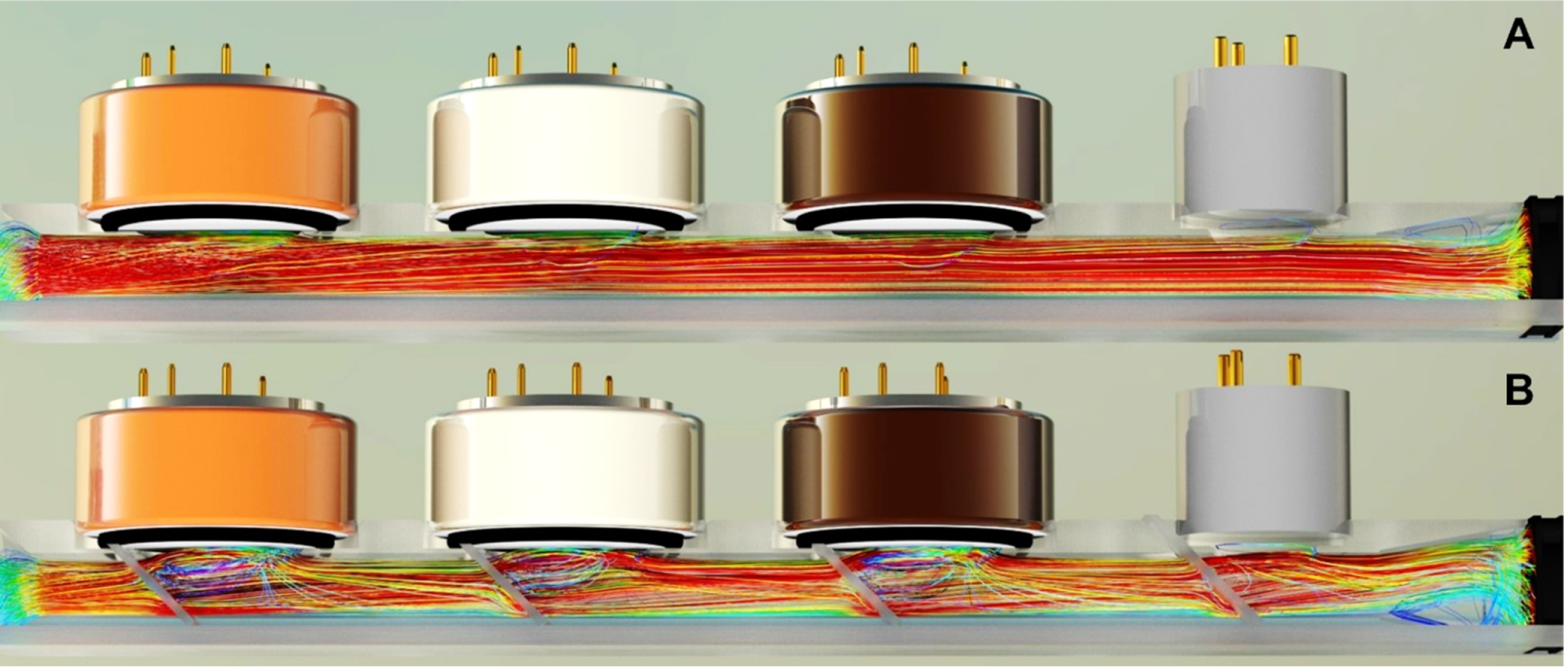
Streamlines through the sensor chamber with and without swirl plates.

### Hardware Description of the Sampling Module

The sampling module is configured for up to 32 industry-standard tubes (31/2” × 1” o.d.), which are placed in a 3D-printed tube holder made from COC. The inlet side of the sorbent tubes are covered with commercially available diffusion-locking caps (DiffLok caps, Markes International Ltd., Bridgend, UK) for preventing sample loss or ingress of airborne substances during storage in the sampler. Furthermore, miniaturized ozone scrubbers were developed for the use in multitube sequential samplers in combination with the DiffLok caps.^20^ These additional scrubbers are necessary for the credible quantification of reactive air contaminations.^21^ To ensure a continuous gas flow of ambient air along the caps, we used a radial fan (Sunon EF50151B3, Sunonwealth Electric Machine Industry Co., Ltd. Kaohsiung, Taiwan), which is mounted on the cover of the case. The air within the sampling module is exchanged at a rate of 20 cycles per minute at a flow rate of 70 L min^–1^.

As can be seen in Figure 2, the ambient air is guided through the case using a radial fan along the inlet side of the sorbent tubes. The air is now sucked through the selected sorbent tube at the predefined flow rate and volume from this air stream. This design ensures that no 3D-printed components or connecting tubes come into contact with the actual sample, as all gas-carrying and regulating components are installed after the airborne pollutants have been adsorbed. Therefore, the inertness of the gas paths is irrelevant for sampling.

**Figure 2:**
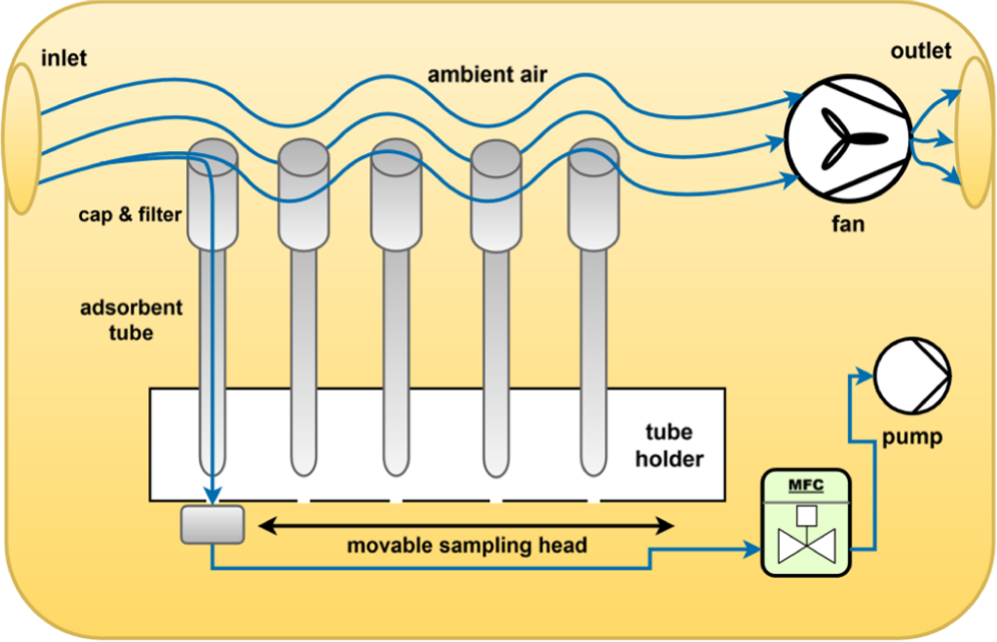
Process diagram of the sampling module.

A 3D cutaway view of the sampling unit is shown in Figure 3. The sorbent tubes are placed vertically in the tube holder (A), and on the surface, a metal plate (B) with stamped position numbers has been attached for mechanical stabilization and to cover the O-rings located underneath. The gas flow through the selected sorbent tubes is realized via integrated gas channels in the lower part of the tube holder (C). The gas channels lead from the position of the sorbent tubes to the center of the module, where the gas outlets of the gas channels are arranged in series. These are now covered precisely by a sealed and movable self-developed sampling head in the middle of the unit (D). The position of this sampling head can be adjusted by a linear actuator (MOT-ST-28-L-A-A, igus GmbH, Cologne, Germany) (E). The sampling head is connected to a mass flow controller (MFC Flexiflow FG-201CV, Bronkhorst High-Tech B.V., Ruurlo, NL) via an inert tube. The differential pressure required for the mass flow controller is generated by a diaphragm pump (SP 270 EC-BLp, Schwarzer, Essen, Germany).

**Figure 3:**
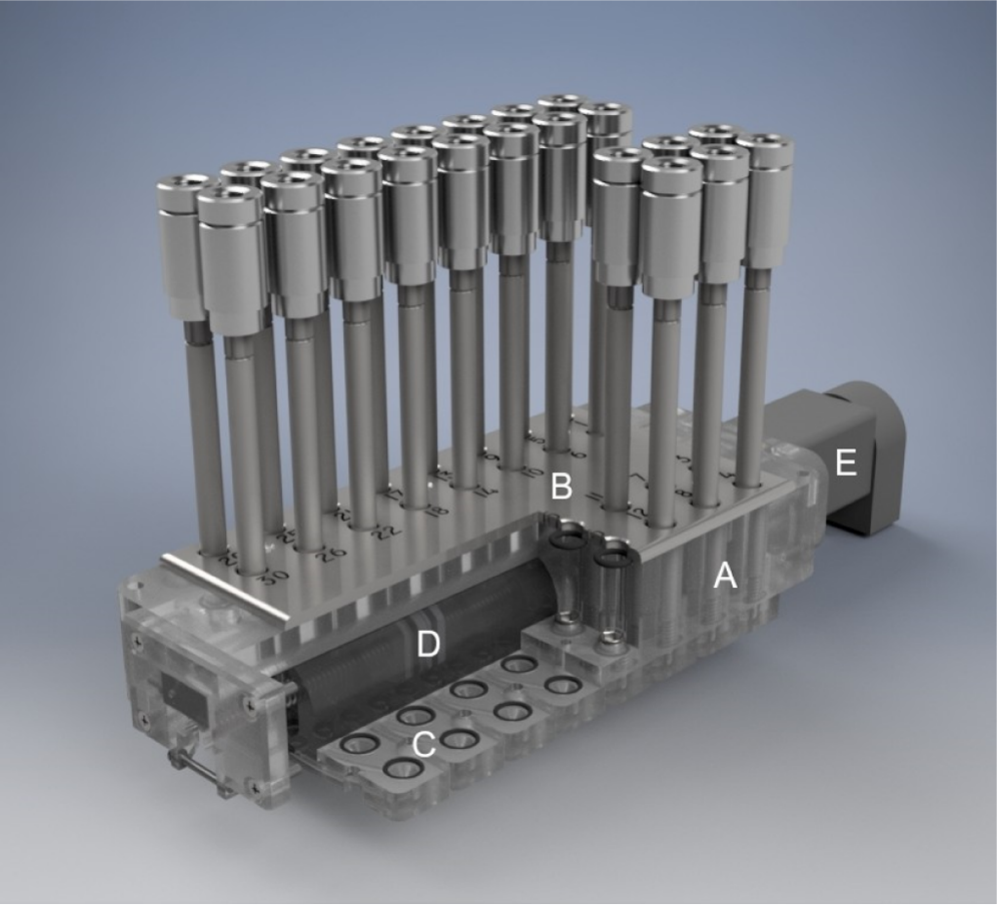
Enrichment unit in the sampling module.

The sample flow can be set in a range between 50 and 500 mL min^–1^. Depending on the adjusted flow rate, an accuracy between ±1% and ±2% can be realized. These deviations result solely from the accuracy of the mass flow controller, as the system was tested for leaks by comparing the inlet and outlet flows.

The sampler module is also equipped with its own control and communication electronics for efficient and autonomous functionality. The in-house-developed electronic module controls all electronic assemblies (sensors, MFC, actuator control, including limit switches) via the corresponding interfaces. Furthermore, it records the measured values of the integrated sensors for inlet pressure of MFC, ambient pressure, temperature within the case, temperature of sample gas flow, volume flow, and total volume. The control electronics are connected to the communication gateway (PRODINo MKR Zero Ethernet, KMP Electronics Ltd., Sofia, Bulgaria) via an RS-485 interface. The gateway is connected to the router in the power module. This approach enables the transfer of sensor data to Raspberry PI single-board computer and the transfer of preselected sample sequence to the sampler module. The parameters (start option and sorbent tube numbers) of the sampling sequence are configured in our web interface.

## Results and Discussion

Although low-cost sensors provide real-time measurements, the quality of the analytical signal is influenced by a number of parameters. In addition to interfering compounds, the sensor signals are also influenced by the temperature and humidity of ambient air. An example is shown in Figure 4 for the calibration of the ozone sensor. The concentration of ozone in the gas stream through the sensor module was adjusted by using an ozone calibration source (Model 306, 2B Technologies, Colorado, USA). Typical gas concentrations of ozone in the environment between 20 and 150 ppb can be easily detected. Parallel calibration curves were observed for two temperatures (25 and 35 °C) of the sensor chamber (Figure 4A). The baseline is slightly lower at higher temperatures, and the slopes and thus the sensitivity are comparable. In practical use, we can minimize temperature influences as much as possible by thermostatting the sensor chamber.

**Figure 4:**
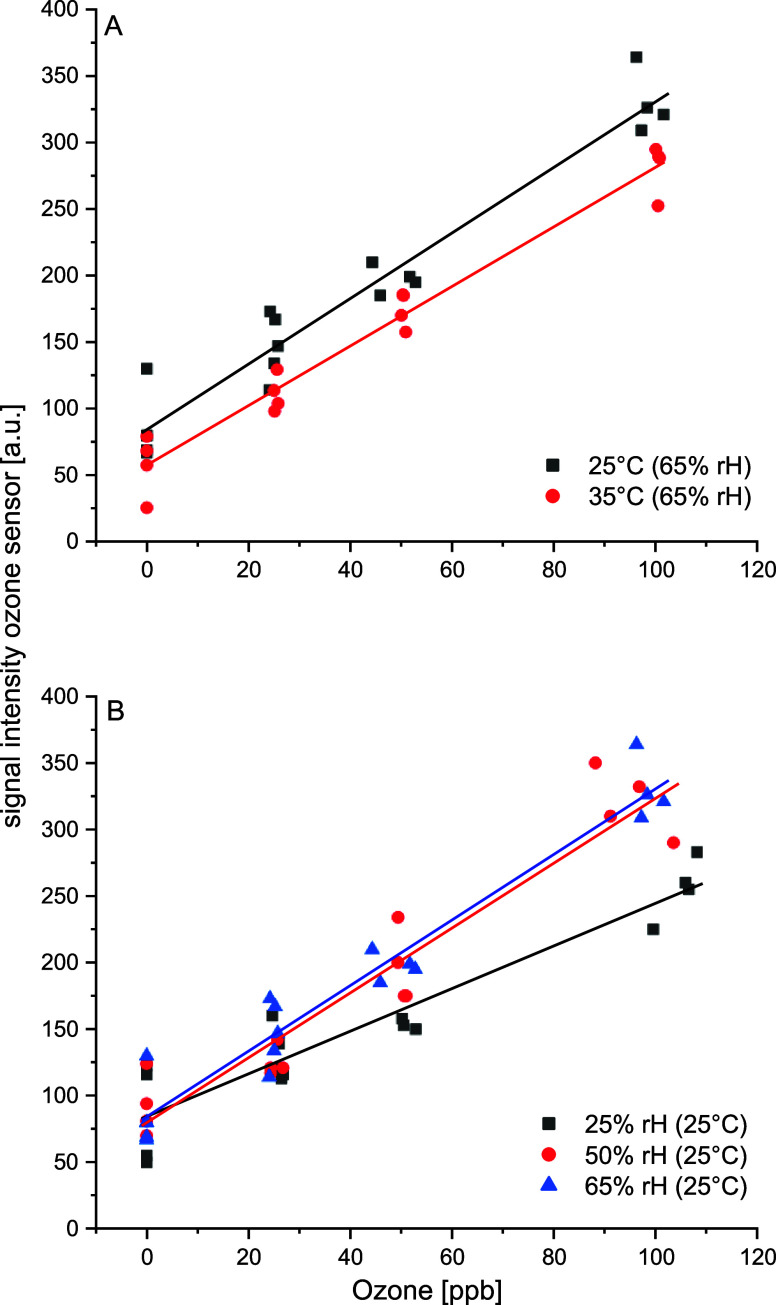
Calibrations of the O_3_ sensor in dependence on (A) temperature and (B) relative humidity (rH).

In contrast to the possible thermostatization of the sensor cell and the resulting minimization of the influence of temperature, the humidity cannot be actively influenced but is permanently recorded during the measurements. The influence on the calibration is relatively low at humidity levels above 50%, the typical range for ambient air (Figure 4B). The height of the baselines is obviously independent of the humidity. However, a strong variance of the values can be observed. A reduced sensitivity can be observed at a lower humidity because the slope of the calibration line is less steep. The analysis of ozone using low-cost sensors requires the parallel determination of nitrogen dioxide due to cross sensitivities. This compensation is integrated into our control software. The NO_2_ sensor was also calibrated depending on temperature and humidity using a Trace Source disposable permeation tube (KIN-TEK Analytical, Inc., La Marque, USA). A comparable temperature and humidity dependence can be observed for the NO_2_ sensor.

Two sensors were used for the determination of the VOCs. The PID can detect substances with an ionization potential below 10.6 eV (mainly unsaturated and aromatic substances), while the electrochemical sensor also detects compounds with higher ionization potentials. In contrast to the sensors mentioned above, the VOC sensors generate sum signals for all detectable substances. Figure 5 shows as an example the calibration of α-pinene using the PID sensor in dependence on humidity. Considering the height of the baseline at 0 ppb, an increase can be observed with increasing humidity. The calibration lines above 50% rH are nearly parallel, while the measurements at 25% rH are more sensitive. However, the required concentrations of single substances are comparatively high (few ppb).

**Figure 5:**
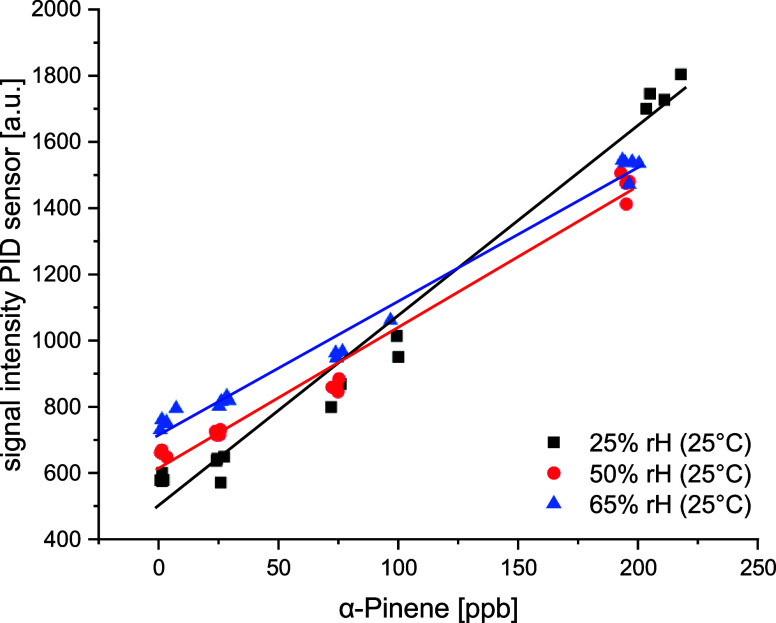
Calibrations of α-pinene using the PID sensor in dependence on relative humidity (rH).

Building on the calibration results of the low-cost sensors, which revealed the influence of the temperature and humidity on sensor signals, we performed a multivariate regression analysis to compensate for these environmental factors. Incorporating temperature and humidity as predictor variables alongside the sensor signals would enable a more robust model to be developed for estimating the concentrations of ozone, nitrogen dioxide, and VOCs.

Consequently, no single compounds can be determined using the VOC sensors at sites with low background concentrations, for example, at forest sites, but since the sum of all detectable organic compounds is displayed, signals can also be obtained at such sites. But this was the purpose of our approach, to use low-cost sensors for determining the presence of organic components in the air and to carry out trace analysis of the individual substances using mass spectrometry.

A key feature of the developed sampler is remote access to all operating parameters. Deviations in the inlet pressure of the mass flow controller indicate technical faults. Depending on the flow resistance of the sorbent tube, the DiffLok cap and scrubber used, a pressure difference from ambient air of 40–70 mbar can be detected. In the case of a leakage in the flow system, these pressure differences decrease below 30 mbar, while a tube clogging leads to pressure differences above 100 mbar. Furthermore, the MFC compensates for differences in pressure and temperature automatically, an important feature for long-term observations in the field.

To maximize flexibility in operation, the system incorporates three distinct sampling modes available for selection within the developed web interface (Figure 6). For event-based sampling, the sensors that will act as event triggers are first selected using switch buttons that are highlighted in blue when activated. Subsequently, specific threshold values are set (in the middle table of the web interface). If the sensor signal exceeds this threshold value for a period of ≥1 min, the system initiates sampling according to the predefined parameters (sample volume, flow rate, etc.). The exact start time and the accompanying sensor signals are recorded in the corresponding log file.

**Figure 6:**
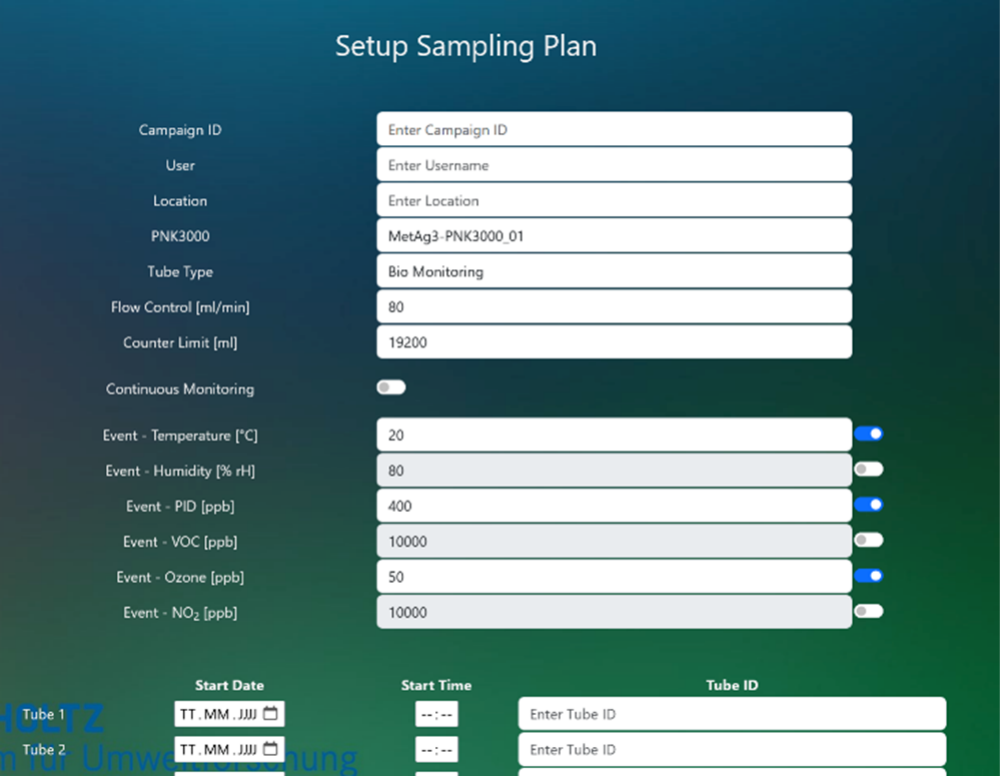
Settings for the start options of the sampler module using the web interface.

The sensor data are continuously logged. The values to be set vary depending on the application and must be determined individually in appropriate calibrations or field tests. It is clear that the different responses of the sensors to different substances lead to different threshold values, for example, for industrial chemicals, for chemical warfare agents, or in environmental monitoring. The sensors can also be replaced according to the applications, which require the adjustment of the threshold values.

Furthermore, an individual start time can be specified for each of the 32 sorbent tubes for time-based sampling (to define in the lower part of the web interface), or continuous monitoring can be started manually. In this case, the sorbent tubes are loaded one after the other. The duration of tube loading is determined by the specified flow rate (Flow Control) and total volume (Counter Limit).

In order to confirm the reproducibility of the sampling module, even across different ambient temperatures, the sampler was placed in a climate chamber and exposed to a gas stream with defined concentrations of benzene (22 μg m^–3^) and α-pinene (5 μg m^–3^), which were produced in a reference gas generator.^22^ Four sorbent tubes at different positions were sampled for 1 h with a gas flow of 80 mL min^–1^ at each temperature. This experiment was performed two times and was carried out using multibed sorbent tubes consisting of Tenax TA and Carbograph 5TD.

The results of this experiment are shown in Figure 7. The column in the diagram shows the mean values obtained from eight replicate sorbent tube samples at each of the four temperature settings employed in the experiment. The standard deviations ranged from 6 to 10%. Furthermore, the efficacy of the system’s temperature compensation is validated by the consistent standard deviations of 8% and 10% for α-pinene and benzene, respectively, when calculated across all samples spanning the entire temperature range.

**Figure 7:**
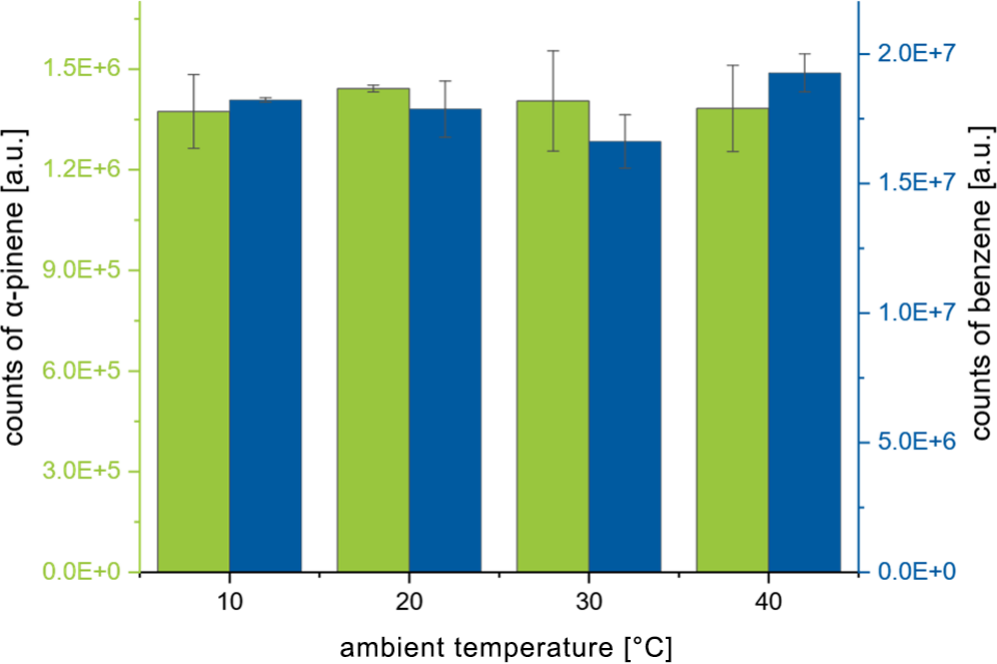
Signal intensities in GC–MS depending on the concentration of ambient air.

The long-term stability during the storage of loaded sorbent tubes was also tested over 12 days (Figure 8). For this purpose, 24 multibed sorbent tubes were loaded with 50 ng standard substances (hexanal, α-pinene, camphene, myrcene, and trans-pinane) and stored in the sampler module with active case fan. The first sorbent tubes were analyzed immediately after loading as a reference (day 1). The sorbent tubes stored in the sampler were analyzed after 5 and 12 days. No additional contamination occurred during storage, and the added substances were found to be highly stable with standard deviations of 4.1%, 2.7%, 1.4%, 2.0%, and 2.4% for hexanal, α-pinene, trans-pinane, and myrcene. It can be summarized that neither additional inputs nor losses were observed during the 12-day storage period.

**Figure 8:**
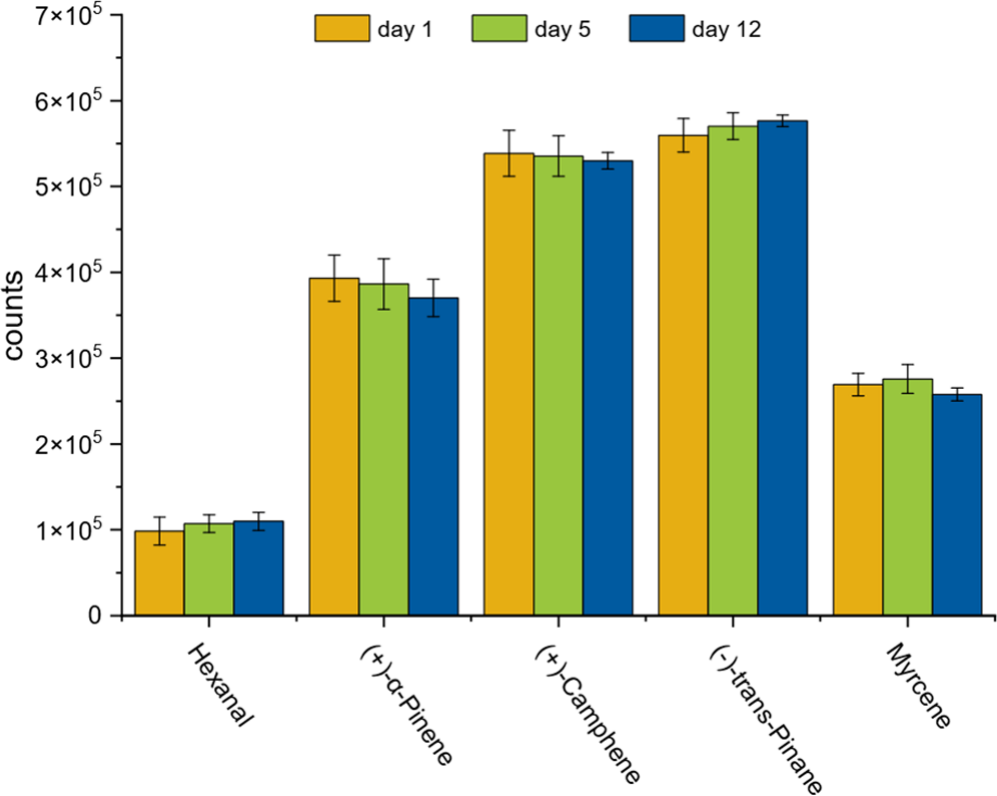
Long-term stability of loaded sorbent tubes in the sampler module.

The air exchange rate of the ambient air sucked into the sampling module was adjusted to be very high in order to ensure a sufficient supply of fresh air and to remove possible emissions from the components of the sampler. Due to the high flow rate of 70 L min^–1^, it is technically difficult to provide purified air in this quantity for recording blanks. For characterization of possible background contamination artifacts arising from the sampler, we compared two different sampling approaches. Two samples were taken in parallel at a distance of a few centimeters. One sample was taken using the sampler. The second sample was sucked directly into the sorbent tube with a diaphragm pump. In both cases, 9.6 L ambient air in an urban area was enriched at a flow rate of 80 mL min^–1^. Each sorbent tube was protected with a DiffLok cap and an ozone scrubber. If the sampler causes background signals, additional peaks should be observed in the chromatogram of the samples taken via the sampler. The results of these measurements are shown in Figure 9.

**Figure 9:**
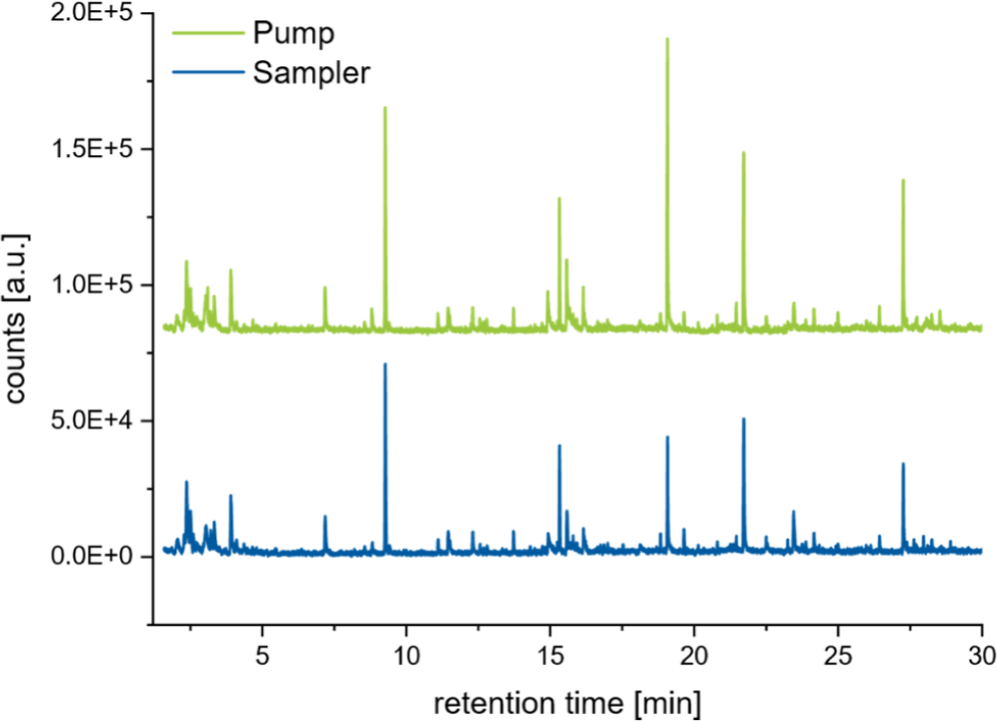
Comparison of chromatograms of two air samples taken in an urban area.

Figure 9 shows nearly identical chromatograms for both samples, and no additional peaks can be observed when using the sampler. Therefore, it can be concluded that the sampler does not cause any significant background signals. Slight differences in intensities may result from the different pumps used for enrichment. In summary, the functionality of the sampler was demonstrated, even though further validation experiments are still necessary.

## Conclusion

The combination of sensors and sampling offers significant advantages for many applications. This means that time-consuming and expensive analysis in the laboratory can be avoided if there is no contamination that can be detected in the environment. The system can be monitored through remote access to all of the essential parameters. Therefore, the operator presence during long-term observations can be reduced. Nevertheless, disturbances during sampling (clogged tubes and deviations in gas flow) can be quickly detected. The entire system can be further adapted for specific applications. Since commercial standard sorbent tubes are used for sampling, the sorbents can be selected according to the sample matrix. The sensor module can also be adapted. More specific sensors for special applications can be used. For example, it is possible to integrate sensors based on ion mobility spectrometry for the monitoring of chemical weapons, which are more sensitive in comparison to standard VOC low-cost sensors. Due to the compact design and autonomous operation, the modules can also be easily mounted on a UGV (unmanned ground vehicles). This allows carrying out measurements with a high spatial resolution via the sensors, and sampling can be activated at locations with high pollutant concentrations. The system developed offers great flexibility for further developments.
